# Phylogenetic analysis and divergence time estimation of *Lycium* species in China based on the chloroplast genomes

**DOI:** 10.1186/s12864-024-10487-9

**Published:** 2024-06-06

**Authors:** Lei Zhang, Erdong Zhang, Yuqing Wei, Guoqi Zheng

**Affiliations:** 1 Key Laboratory of Ecological Protection of Agro-Pastoral Ecotones in the Yellow River Basin, College of Biological Science & Engineering, National Ethnic Affairs Commission of the People’s Republic of China, North Minzu University, Yinchuan, 750021 China; 2 Key Laboratory of the Ministry of Education for Protection and Utilization of Special Biological Resources in the Western, School of Life Science, Ningxia University, Yinchuan, Ningxia 750021 China

**Keywords:** *Lycium*, Plastome structure, Comparative analysis, Phylogenetic relationship, Divergence time

## Abstract

**Background:**

*Lycium* is an economically and ecologically important genus of shrubs, consisting of approximately 70 species distributed worldwide, 15 of which are located in China. Despite the economic and ecological importance of *Lycium*, its phylogeny, interspecific relationships, and evolutionary history remain relatively unknown. In this study, we constructed a phylogeny and estimated divergence time based on the chloroplast genomes (CPGs) of 15 species, including subspecies, of the genus *Lycium* from China.

**Results:**

We sequenced and annotated 15 CPGs in this study. Comparative analysis of these genomes from these *Lycium* species revealed a typical quadripartite structure, with a total sequence length ranging from 154,890 to 155,677 base pairs (bp). The CPGs was highly conserved and moderately differentiated. Through annotation, we identified a total of 128–132 genes. Analysis of the boundaries of inverted repeat (IR) regions showed consistent positioning: the junctions of the IRb/LSC region were located in *rps*19 in all *Lycium* species, IRb/SSC between the *ycf*1 and *ndh*F genes, and SSC/IRa within the *ycf*1 gene. Sequence variation in the SSC region exceeded that in the IR region. We did not detect major expansions or contractions in the IR region or rearrangements or insertions in the CPGs of the 15 *Lycium* species. Comparative analyses revealed five hotspot regions in the CPG: *trn*R(UCU), *atp*F-*atp*H, *ycf*3-*trn*S(GGA), *trn*S(GGA), and *trn*L-UAG, which could potentially serve as molecular markers. In addition, phylogenetic tree construction based on the CPG indicated that the 15 *Lycium* species formed a monophyletic group and were divided into two typical subbranches and three minor branches. Molecular dating suggested that *Lycium* diverged from its sister genus approximately 17.7 million years ago (Mya) and species diversification within the *Lycium* species of China primarily occurred during the recent Pliocene epoch.

**Conclusion:**

The divergence time estimation presented in this study will facilitate future research on *Lycium*, aid in species differentiation, and facilitate diverse investigations into this economically and ecologically important genus.

**Supplementary Information:**

The online version contains supplementary material available at 10.1186/s12864-024-10487-9.

## Background

*Lycium* is a crucial shrub genus in the Solanaceae family, consisting of approximately 70 species found globally across Southern Africa, Europe, Asia, America, and Australia [1, 2]. However, China hosts 15 species, including subspecies, primarily growing in the arid and semi-arid regions of Ningxia, Xinjiang, Inner Mongolia, and other areas [3–7]. These plants have been utilized as food and herbal medicine in China for millennia [8, 9], with various parts like fruits, leaves, root bark, and young shoots used as local foods and/or medicines [10]. These plants impart potential pharmacological effects such as anti-aging properties, reduction of blood glucose and serum lipids, and immune regulation [11]. Several studies have demonstrated that extracts of these plants can prevent and treat diseases such as night sweats, diabetes, cough, vomiting, hypertension, and ulcers [10, 12, 13]. Overall, previous studies have focused on the growth, development, medicinal value, and breeding of *Lycium* [14–17]. Despite its economic and ecological significance, much remains unknown about *Lycium* phylogeny, interspecific relationships, and evolutionary history. While numerous studies have identified and analyzed phylogenetic relationships within the *Lycium* genus using DNA barcode fragments [18–22], research on CPG phylogeny and lineage diversification is lacking.

Chloroplasts are essential organelles involved in photosynthesis and carbon fixation in plant cells. The advancement in sequencing technologies has made whole CP sequencing more accessible [23–25]. Compared with DNA fragments, CPGs contain significantly more informative sites for analyzing nucleotide diversity and reconstructing phylogenies among closely related species [26–28]. CPGs typically range from 75 to 250 kb in length, with numerous copies in a given cell, are maternally inherited in most plants, and have conserved gene content and order [29, 30]. The plastome is characterized by two large inverted repeat regions (IRa and IRb) separated by two single-copy regions, referred to as the large single-copy region (LSC) and the small single-copy region (SSC) [31]. Recently, CPGs have been used for comparative and phylogenetic analyses, proving useful in species identification, genetic diversity assessment, nucleotide diversity assessment, resolving phylogenetic relationships, and evolutionary history [32–36].

Despite the use of CPGs, few molecular phylogenetic studies have attempted to resolve infrafamilial relationships within *Lycium* using broad taxon sampling. Most of these studies were based on one or a few molecular loci or had sampling limitations, leaving our understanding of the phylogenetic relationships and divergence timescales of the genus unclear [18, 37–39]. In this study, we sequenced and aligned the CPGs of 15 species, including subspecies, of the genus *Lycium* from China. Our main objectives were to (a) construct a phylogeny based on the CPGs of 36 species from the Solanaceae family; (b) date the divergence of the *Lycium* clade; and (c) examine structural changes in the CPGs of the sampled *Lycium* species.

## Results

### Characteristics of *Lycium* chloroplast genomes

**Fig. 1:**
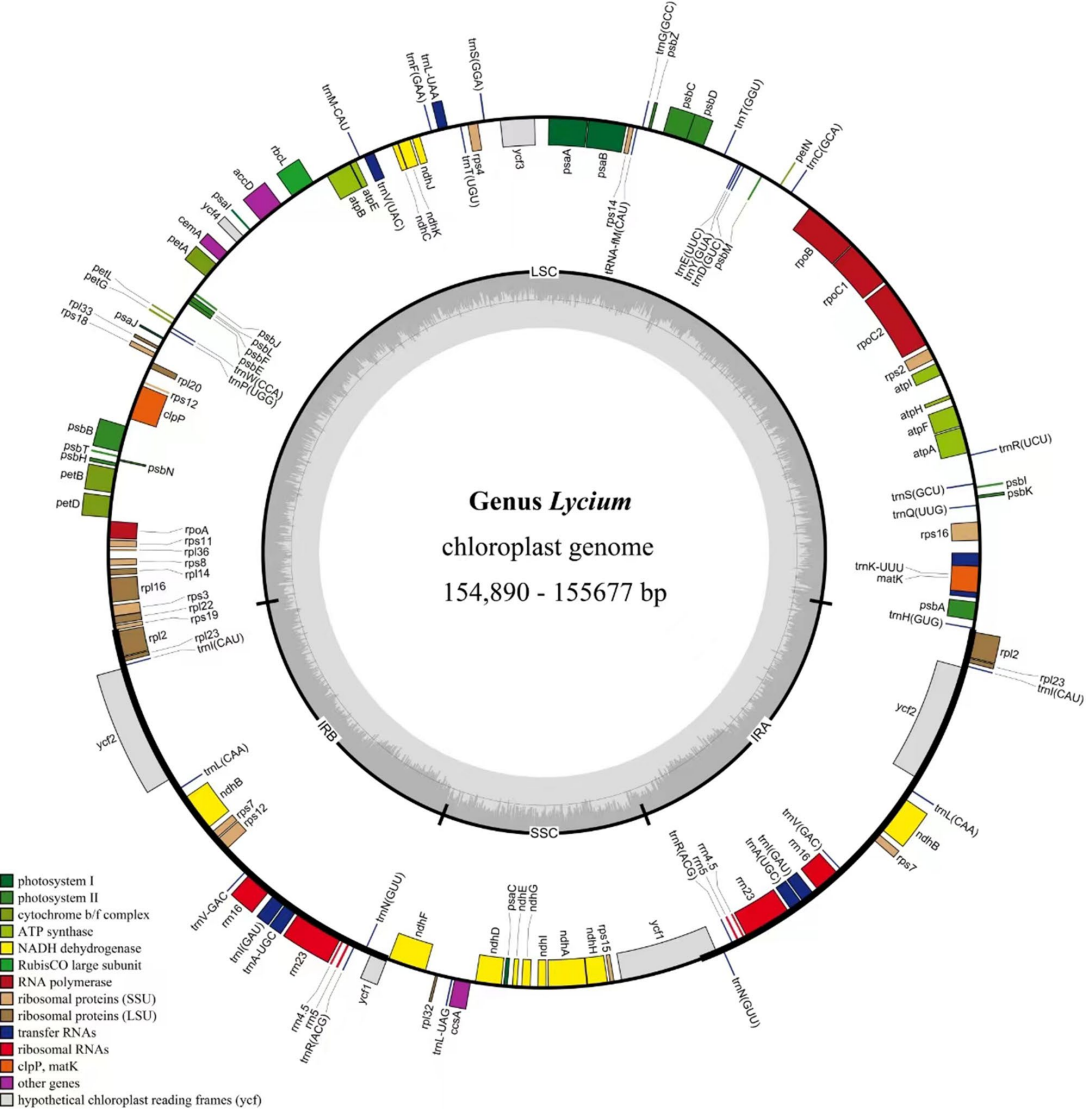
Gene map of the CPGs of fifteen *Lycium* species. Genes belonging to different functional groups are shown in different colors. The darker gray area in the inner circle indicates the GC content and the lighter gray indicates the AT content of the genome. The thick lines indicate the extent of the inverted repeats (IRa and IRb) that separate the genomes into the small single-copy (SSC) and large single-copy (LSC) regions

After quality control and pre-processing, a minimum of 6 Gb of whole-genome sequencing data were obtained for each of the 16 species included in this study (Table S1). These clean reads were used to assemble complete CPs using a reference-guided approach. All the newly assembled CPGs displayed a typical quadripartite structure, with two IR regions separating the LSC and SSC regions (Fig. 1).

For each of the 15 *Lycium* species, the CPGs size ranged from 154,890 bp (*L. changjicum*) to 155,677 bp (*L. amarum*) (Table S1). All the CPGs had a typical quadripartite circular structure (Fig. 1) consisting of an LSC and an SSC region separated by a pair of IR regions (Fig. 1 and Table S1). The LSC region’s length varied from 85,892 bp (*L. changjicum*) to 86,635 bp (*L. amarum*), and the lengths of the SSC and IR regions ranged from 18,190 bp (*L. cylindricum*) to 18,215 bp (*L. barbarum*) and 25,394 bp (*L. ruthenicum*) to 25,469 bp (*L. cylindricum*), respectively (Table S1). The GC content of the entire plasmid sequence and the LSC, SSC, and IR regions was similar across all *Lycium* CPGs. Specifically, the GC content of the entire plasmid sequence was 37.8–37.9%; while the GC content of the IR regions was 43.1–43.2%, which was higher than that of the LSC and SSC regions (35.8–35.9% and 32.3–32.4%, respectively; Table S1). Additionally, the number of annotated genes in each CPGs ranged from 128 (*L. amarum*) to 132 (*L. ningxiaens*), and included 35–37 tRNA and 8 rRNA genes.

### Comparative genomics and divergence hotspots

**Fig. 2:**
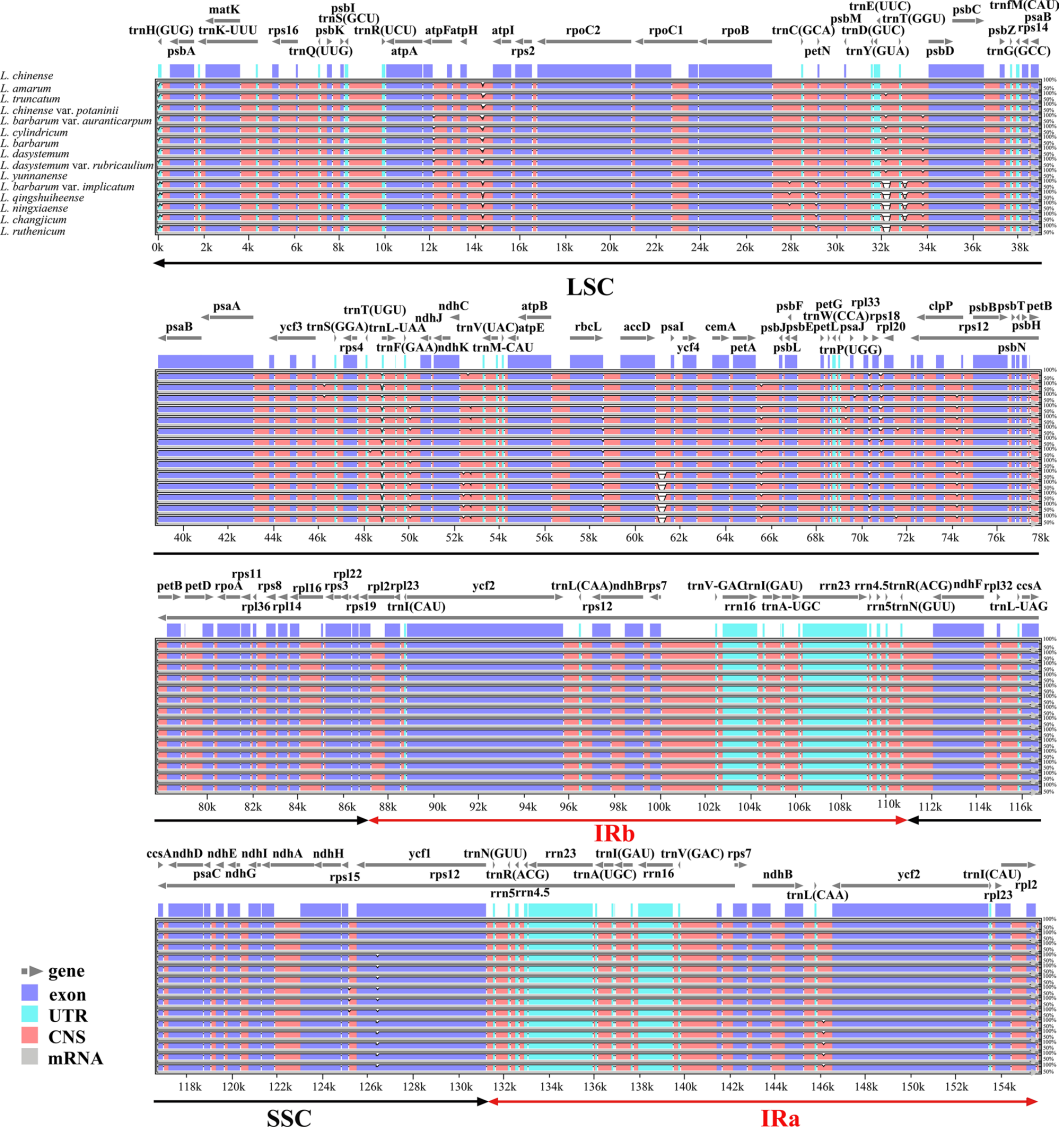
Sequence alignment of the CPGs of *Lycium* species. The alignment was performed using the mVISTA program and the *L. chinense* chloroplast genome was used as a reference. The Y-axis: the degree of identity ranging from 50 to 100%. Coding and non-coding regions were marked in blue and red, respectively. Black arrows indicated the position and direction of each gene. CNS: conserved non-coding sequences

**Fig. 3:**
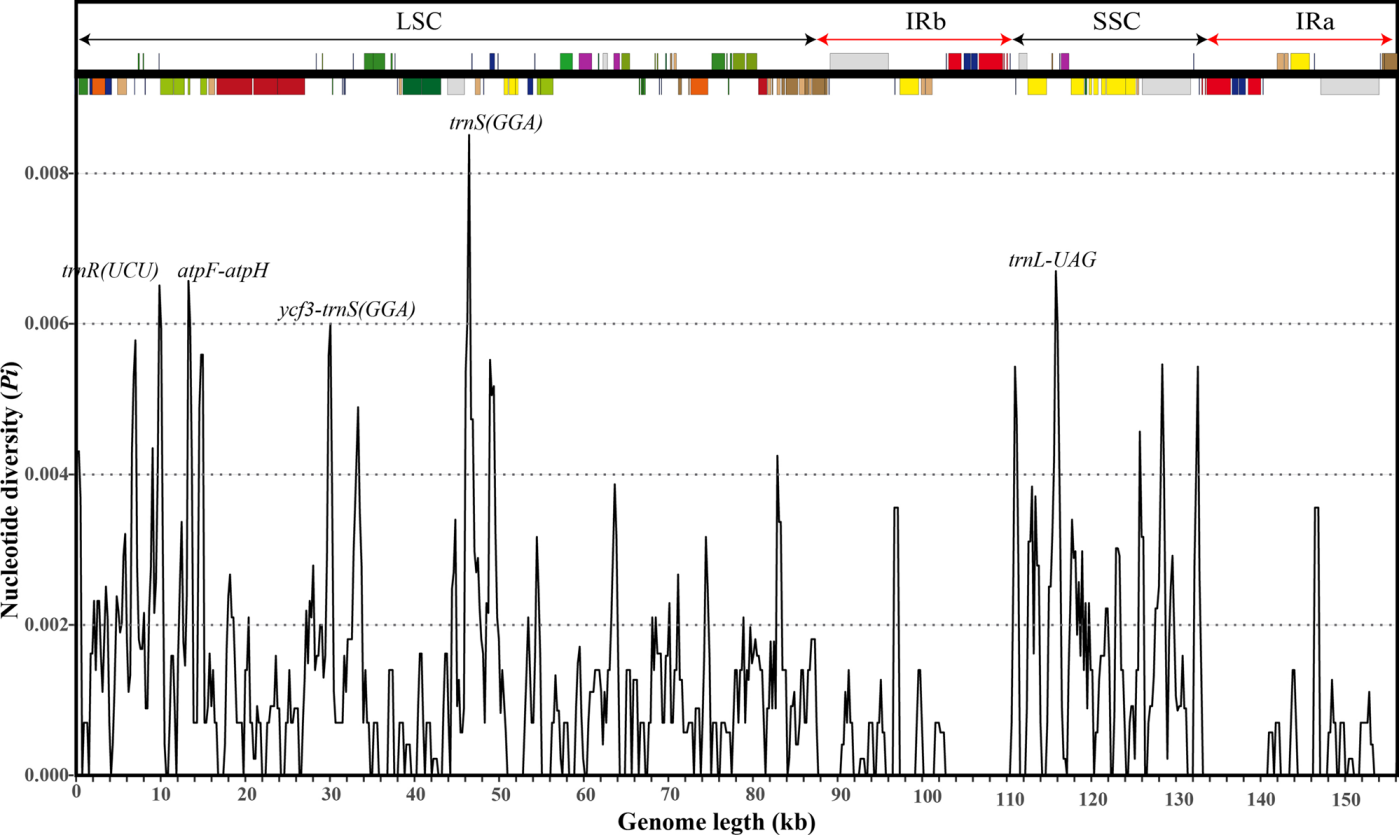
Sliding window test of nucleotide diversity (Pi) in the multiple alignments of 15 *Lycium* species (window length: 600 bp; step size: 200 bp). X-axis: the position of the midpoint of the window; Y-axis: the nucleotide diversity of each window

Using *L. chinense* as a reference, the CPGs of the 15 *Lycium* species were visually compared with those obtained using the mVISTA online database. The results showed that the CPGs of the 15 species were conserved, especially the coding regions (Fig. 2). Considering the entire plastome, the SSC and LSC regions displayed marked divergence compared to the IR regions. The proportion of non-coding regions was greater than that of protein-coding regions, and divergence hotspots were largely located in the intergenic spacer regions (Fig. 2). These divergence hotspots usually consist of highly variable sequences that can be used as potential DNA barcodes for phylogenetic analyses and to determine relationships between species. Therefore, to further understand DNA polymorphisms (Pi), mutation hotspot regions in the CPGs of the 15 *Lycium* plants were screened using DnaSP software (Fig. 3). Pi analysis revealed that Pi values ranged from 0 to 0.00851, and the CPGs were relatively structurally conserved, small, and highly variable among the species. We identified five mutation hotspot regions (Pi > 0.0006) that could be used as potential molecular markers. Of these, *trn*R(UCU), *atp*F-*atp*H, *ycf*3-*trn*S(GGA), and *trn*S(GGA) are located in the LSC region, while *trn*L-UAG is located in the SSC region (Fig. 3). None of the mutation hotspots are located in the IR region.

**Fig. 4:**
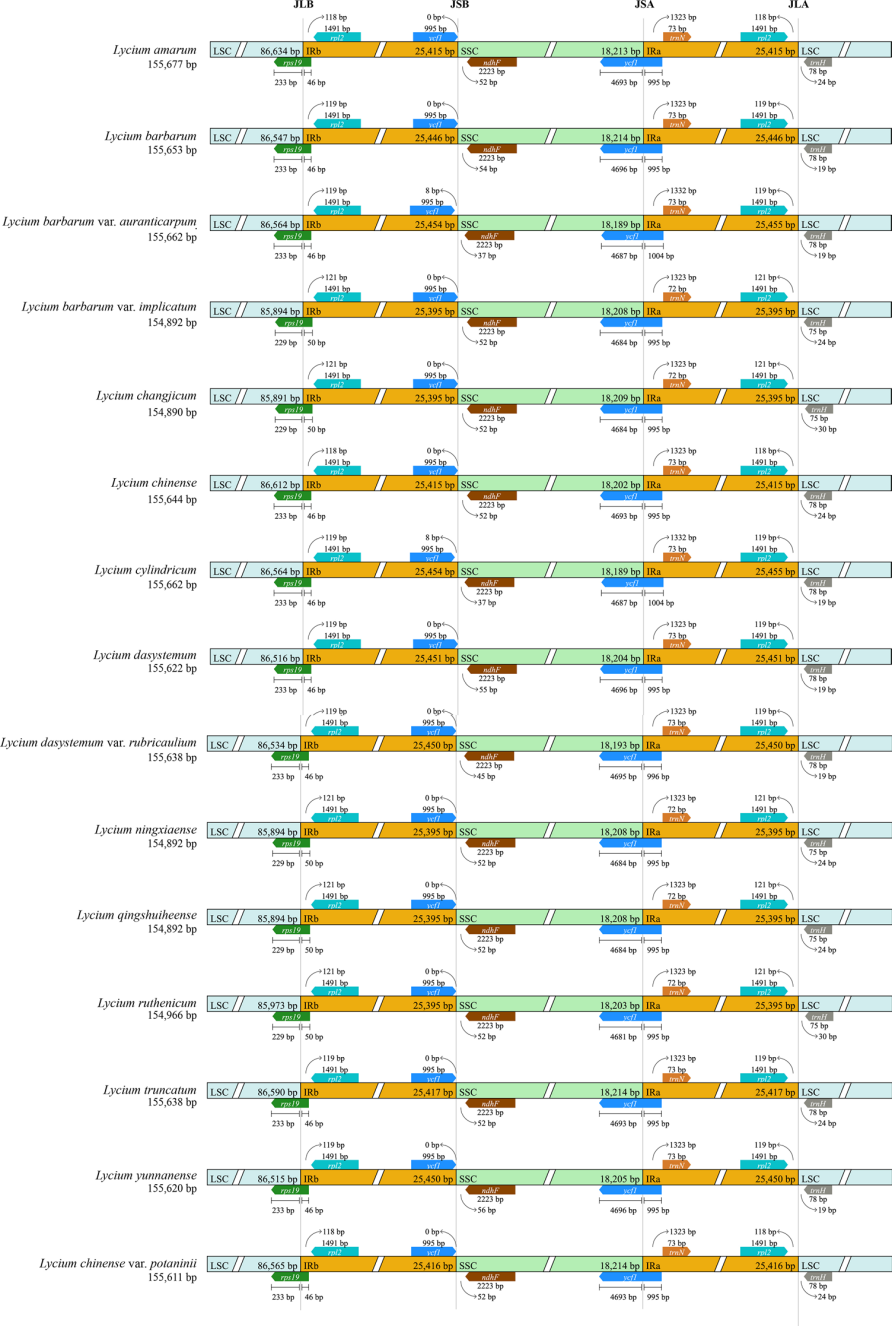
Comparisons of the borders of the large single-copy (LSC), small single-copy (SSC), and inverted repeat (IR) regions among the CPGs of fifteen *Lycium* species

### Boundaries between IR and SC regions

The boundaries of the LSC, SSC, and IR regions were highly consistent within *Lycium* and no obvious expansion or contraction of the IR region was detected in the 15 CPGs (Fig. 4). Here, *trn*H was shown to be the first gene in the LSC region at the junction between IRa and LSC (i.e., IRa/LSC). At the other end of the LSC region, the IRb/LSC junctions were located in the *rps*19 gene sequence in all *Lycium* species, with the length of the *rps*19 gene located in the IRb region, varying from 46 to 50 bp. At both ends of the SSC region, IRb/SSC junctions were located between the *ycf*1 and *ndh*F genes. However, SSC/IRa was found in the *ycf*1 gene, with the length of the *ycf*1 gene located in the IRa region varying from 995 to 1004 bp.

### Phylogenetic analyses of Solanaceae

**Fig. 5:**
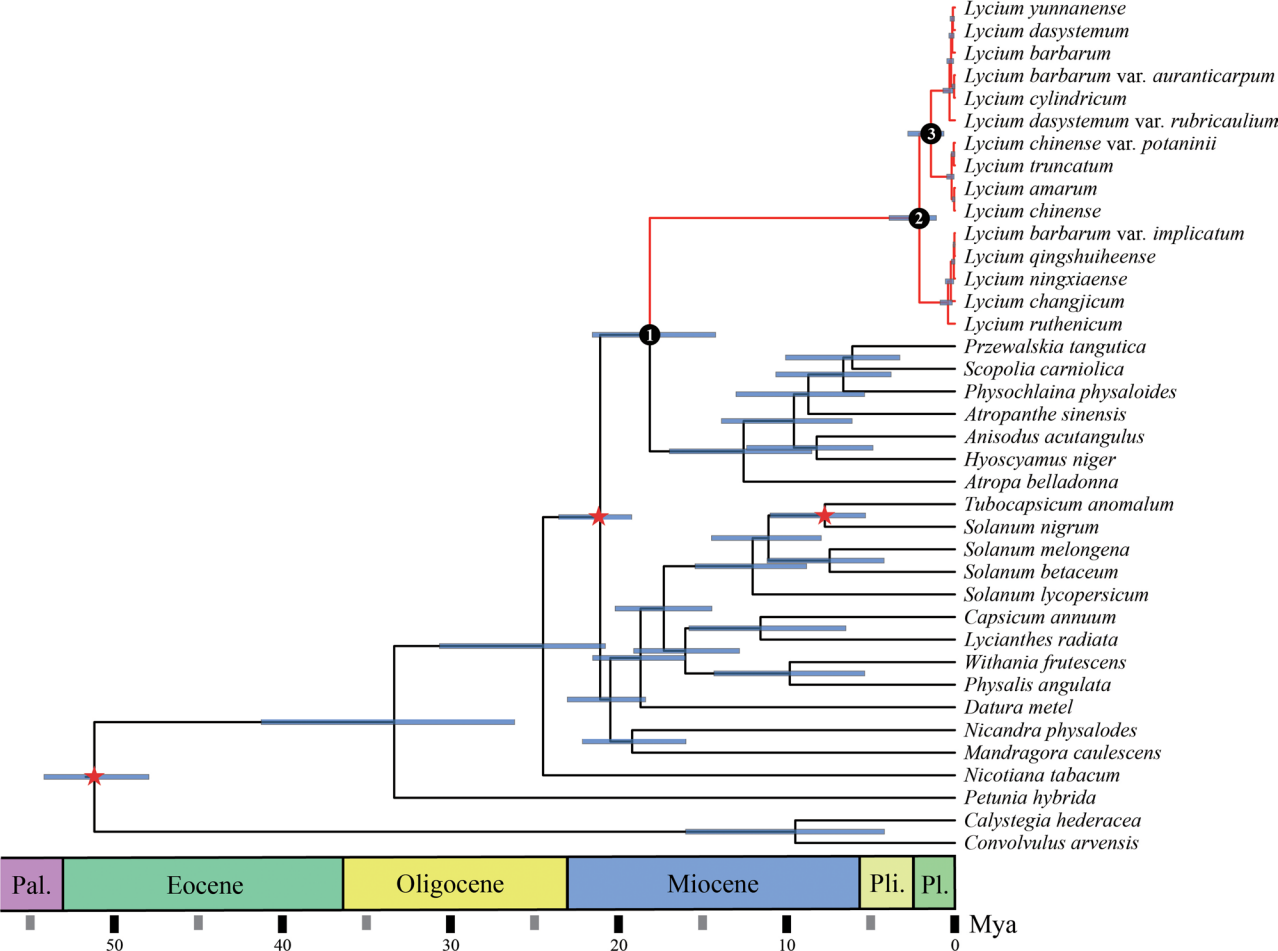
Phylogeny and clade divergence of Solanaceae and outgroups based on 55 plastome protein-coding genes. Stars indicate fossil and time constrations in this analysis. Geological periods are marked with background colors. Mya: million years ago; Pal: Paleocene; Pli: Pliocene; Ple: Pleistocene

To infer the phylogenetic relationships of the 36 Solanaceae species, we included two Convolvulaceae species whose CPGs are publicly available in the GenBank database. These species (*Calystegia hederacea* and *Convolvulus arvensis*) were used as the outgroup for phylogenetic analyses. The final concatenated dataset included 55 plastid genes and 42,960 sites, of which 2992 were parsimony informative, after trimming poorly aligned regions and gaps with missing genes (Table S2). In the phylogenetic trees, most nodes in the ML and BI analyses of each dataset generated almost congruent topologies with high bootstrap support (BS > 90%) and posterior probability (PP = 1) values (Fig. 5 and S1, S2, S3, S4). Thirty-six plant species belonging to the Solanaceae family were divided among three typical branches. Branches of *Physochlaina physaloides*, *Przewalskia tangutica*, *Scopolia carniolica*, *Atropanthe sinensis*, *Solanum betaceum*, *Anisodus acutangulus*, *Hyoscyamus niger*, and *Atropa belladonna* were resolved as sisters of *Lycium*. Further, in the CDS phylogeny, *Lycium* species were clustered on one large branch, confirming that the independence of this genus is highly supported (BS; PP = 100%, 1) (Fig. S1 and S2). The 15 species of plants belonging to *Lycium* were divided into two typical sub-branches and three minor branches: clade I-1, comprising *L. barbarum* var. *auranticarpum*, *L. cylindricum*, *L. yunnanense*, *L. barbarum*, *L. dasystemum* var. *rubricaulium*, and *L. dasystemum* on one sub-branch (BS; PP = 100%, 1); clade I-2 consisting of *L. chinense* var. *potaninii*, *L. truncatum*, *L. amarum*, and *L. chinense* on another sub-branch (BS; PP = 100%, 1), and clade II comprising *L. barbarum* var. *implicatum*, *L. qingshuiheense*, *L.ningxiaense*, *L. changjicum*, and *L. ruthenicum* on a different branch (BS; PP = 100%, 1).

**Table 1:** Estimated ages for *Lycium* subclades

Subclade name^*^	Mean age (Mya)	95% highest posterior density interval (HPD)
Subclade 1	17.7	14.0-21.3
Subclade 2	4.95	1.09–3.86
Subclade 3	1.7	0.64–2.76

*Subclades are labeled in Fig. 5

### Divergence time estimation

We estimated the divergence timescales of the major clades within *Lycium* according to the calibration of the gene tree constructed based on 55 plastid genes. The split between *Lycium* and its sister group was estimated to have occurred 17.7 Mya. The crown ages of all subclades in the genus *Lycium* were dated mainly within the Pleistocene, suggesting that numerous species of this genus originally diversified in the recent past (6 Mya) (Fig. 5; Table 1).

## Discussion

Recent several studies have reported that most angiosperms have CPGs ranging in size from 120 to 170 kb, with the IR region typically spanning 20–30 kb [16, 27, 28]. In the present study, a comparative analysis of the CPGs indicated that those of 15 *Lycium* species from China were at the larger end of the spectrum for angiosperm organelle genomes, ranging from 154,890 bp (*L. changjicum*) to 155,677 bp (*L. amarum*). All *Lycium* species exhibited a typical quadripartite structure that is similar to other vascular plants, comprising the LSC region (85,892–86,635 bp), SSC region (18,190–18,215 bp), and two identical IR regions (25,394–25,469 bp). The GC content of the entire plastid sequence and the LSC, SSC, and IR regions was similar across all *Lycium* CPGs. Consistent with the results of numerous studies, the GC content of the IR region is relatively the highest among the four chloroplast genome regions [27], potentially explaining the conservation of the IR region [28, 29]. However, the IR partition shrinkage and expansion of the edge were the main factors of the size change, also enabling easy pseudogene production [34, 35]. In our study, we analyzed 15 CPGs within the highly conserved *Lycium* species and noticed that no major expansions or contractions occurred in the IR regions.

Highly variable regions offer valuable phylogenetic information. For example, variable regions aid can be used as development of molecular markers and species delimitation [38–41]. A good molecular marker must be a short, representative DNA fragment with high variability and amenable to amplification [42]. In this study, both the sequence and structure of *Lycium* CPGs were highly conserved. mVISTA analysis revealed that most of the variation in nucleotide sequences occurred in noncoding regions, consistent with previous reports, suggesting this variation as a common feature of angiosperms [43–45]. In the *Lycium*, several highly variable regions, such as *mat*k, *rps*16–*trn*K and *trn*H–*psb*A, are recognized as potential DNA barcoding sites [39, 46]. In addition, our nucleotide diversity (Pi) analysis led to the identification of five highly variable regions with Pi values greater than 0.006, including three non-coding tRNA regions (*trn*S, *trn*L, and *trn*R) and two intergenic regions (*atp*F-*atp*H and *ycf*3-*trn*S (GGA). In conclusion, these mutation hotspot regions play an important role in the identification and characterization of *Lycium* plant species.

*Lycium* is a genus of shrubs with significant economic and ecological importance. Therefore, considerable efforts have been dedicated to cultivated practices, growth and development, medicinal application, and breeding [10–17]. However, to date, few studies have explored its phylogenetic relationships and divergent timescales [11]. The CPGs are central to molecular biology research and have become a prominent focus. In recent years, due to advances in sequencing technology and reduced costs, whole-genome sequencing has become an important tool for species identification, phylogenetic relationships, and evolutionary history reconstruction. Single- or several-gene-segment-based phylogenetic trees often produce inconsistent or even conflicting topologies due to the evolutionary rates and gene introgression. This complexity affects precise evolutionary relationship determination between species [47, 48]. In this study, we constructed a phylogenetic tree using the BI and ML methods. The CPGs of 15 *Lycium* species converged into branches with high support (Fig. S3 and S4). Notably, *L. cylindricum*, *L. yunnanense*, *L. dasystemum*, and *L. dasystemum* were similar to *L. barbarum* and closely related, while *L. chinense* was classified in the same branch as *L. truncatum* and *L. amarum* (BS; PP = 100%, 1.00, respectively), indicating that the three species are similar. Based on the phylogenetic tree results, we speculate that *L. qingshuiheense*, *L. ningxiaense*, and *L. changjicum* may have originated from *L. ruthenicum*. Although *L. barbarum* var. *implicatum* and *L. qingshuiheense* formed a small, distinct branch, the support rate was low, indicating that they may be the same species. This study demonstrates that high-resolution CPG sequences provide valuable resources for broad research on genetic information and species identification of *Lycium* spp.

The highly conserved and stable alignment of the 55 plastid genes allowed us to calibrate the divergence and origin of *Lycium* (Fig. 5). We used three tentative calibrations to estimate the diversification. While the estimated ages should be used with caution, our findings indicate that the *Lycium* diverged from the sister genus around 17.7 Mya, and the two successive clades within the *Lycium* diverged 4.95 and 1.7 Mya, suggesting relatively late clade diversifications. Specifically, most species diversification within the subclades of *Lycium*, as estimated from these plastid genes, appeared to have occurred in the recent past, mostly after 5 Mya., despite the fact that numerous species are currently acknowledged in genera. This observation may partly account for the widespread hybridization observed among these young species [46], likely resulting from incomplete reproductive isolation. The divergence timescales estimated here for the major subclades will serve as a basic timescale for diverse studies on this economically and ecologically important genus.

## Conclusions

We analyzed the complete CPGs of 15 *Lycium* species and found that all exhibited a quadripartite structure, typical of most angiosperms. The CPG arrangement was highly conserved, with great sequence variation observed in the SSC region compared with that in the IR region. We did not detect major expansions or contractions in the IR region, nor did we find any rearrangements or insertions in the CPGs of the 15 *Lycium* species. We identified highly variable regions within *Lycium* that are likely to be useful for species delimitation. Phylogenetic tree construction based on the CPGs showed that all 15 *Lycium* species formed a monophyletic group, divided into two typical subbranches and three minor branches. Molecular dating suggested that *Lycium* diverged from its sister genus around 17.7 Mya, with species diversification of the *Lycium* species of China occurring mainly within the recent Pliocene epoch. Overall, our findings and the estimated divergence times will facilitate future studies on *Lycium*, assist in species differentiation, and facilitate diverse studies of this economically and ecologically significant genus.

## Materials and methods

### Taxon sampling, DNA extraction, and plastome sequencing

A total of 38 CPGs representing Solanaceae and related families were included in this study (Table S3). 36 CPGs from Solanaceae were selected, including all *Lycium* species found in China. Further, two additional CPGs from related families in Convolvulaceae were chosen as outgroups for phylogenetic analysis. Among these 38 CPGs, 16 complete CPGs were newly sequenced, and the others were obtained from GenBank (Table S3). The leaves used in this study were collected from natural populations in China (Table S3). The plant materials were identified by Dr. Lei Zhang and the voucher specimens (Table S2) were deposited in the Herbarium of North Minzu University (NMU; Yinchuan, China). For each species, we extracted total DNA from dried leaves preserved them in silica gel using the CTAB protocol [49]. Paired-end libraries with an insert size of 500 base pairs (bp) were constructed by Illumina (Qingdao, Shandong, China) following sequencing with a HiSeq × Ten System (Jizhi, Qingdao, Shandong, China).

### Chloroplast genome and annotation

At least two gigabases (Gb) of 2 × 150 bp short read data were generated for each sample. Reads with quality scores of less than 7 and with more than 10% ambiguous nucleotides were filtered. The remaining reads were assembled using NOVOPlasty version 2.7.2 [50] software with k-mer = 39, read length = 150 and insert size = 350. The contigs were aligned into sequence in Geneious version 9.1.8 [51] software using the *L. chinense* CPG as a reference. The CPGs were annotated using Plann version 1.1 [52]. Protein-coding genes were extracted using customized Python scripts. Alignment of chloroplast genes across all species was performed using PRANK version 130,410 [53] software using the codon model. Poorly aligned regions were trimmed using Gblocks version 0.91b [54] with the option “−t = c,” selected to set the type of sequence to codons. Genes that were absent in at least one species were excluded, and the aligned sequences were combined into a super matrix. Additionally, circular maps of the CPGs were created using OGDRAW version 1.2 [55], and all annotated CPGs were submitted to GenBank [56].

### Comparative genomics and structural analyses

The structural variation and identification of arrangement events across *Lycium* was conducted for the 15 CPGs of *Lycium*. The results of the comparative analysis of the CPGs were visualized with the mVISTA program [57] and the annotated CPG of *L. chinense* was used as the reference in the LAGAN mode [58]. The junction sites of the four structural regions (IRA, LSC, SSC, and IRB) and adjacent genes in 15 *Lycium* CPGs were visualized and compared using IRscope [59] software to obtain a macroscopic view of the CPG structure. Following sequence alignment, nucleotide diversity (Pi) analysis of the CPG was performed using DnaSP version 6.0 [60].

### Phylogenetic inference and divergence time estimation

We generated two datasets for phylogenetic analysis: a protein-coding region (CDS) set and a whole plastome (WP) set. Protein-coding genes (PCGs) were extracted from the GenBank formatted file containing 38 CPGs using customized Perl scripts that removed the start and end codons. After excluding possible pseudogenes, 55 PCGs were retained in all species. Each PCG was aligned using PRANK version130410 based on the translated amino acid sequences. Genes that had been lost in at least one species were discarded and then the remaining aligned sequences were concatenated into a super matrix. Independent phylogenetic analyses were performed for each dataset (CDS and WP) using the maximum likelihood (ML) and Bayesian inference (BI) methodologies. We used RAxML version 8.1.24 [61] to conduct ML analyses with a general time reversible model with a gamma distribution (GTR + Γ). The best-scoring ML tree was obtained using the rapid hill-climbing algorithm (i.e., the option “-f d”) with 1,000 bootstrap replicates. The optimal model (GTR + I + G) was identified using jModeltest software, and BI analysis was conducted using MrBayes version 3.2.6 [62]. Additionally, FigTree version 1.4.2 [63] was used to visualize phylogeny.

We estimated divergence times from the plastome dataset using an approximate likelihood method, as implemented in MCMCtree in PAML version 4 [64] software, with independent relaxed-clock and birth–death sampling [65] strategies. Fossil dates and time constrations were used as calibration points to reduce bias for more accurate age estimates [66]. However, the fossil records of Solanaceae are very limited. In order to estimation the divergence time of *Lycium*, we choosed one fossil and two other time constrations to calibrate the phylogeny: (1) According to the ancient seed fossil of *Solanum nigrum* [67], 5.3–11.6 Mya was assigned to the split between *Solanum nigrum* and its sister species *Tubocapsicum anomalum*. (2) The split between *Solanum* and *Lycium* was assigned an age range of 19.0-23.3 Mya as previously estimated [68]. (3) The root of the phylogeny was restricted to an age range of 46.2–53.7 Mya based on the secondary age constraints described by Särkinen et al. [68]. The best-fit GTR + Γ model was selected and the prior of the substitution rate (rgene) was modeled by a Γ distribution as Γ (2, 200, 1). We set parameters for the birth–death process with species sampling and σ^2^ values of (1, 1, 0.1) and G (1, 10, 1), respectively. We executed the MCMC runs for 2,000 generations as burn-in and then sampled every 750 generations until 20,000 samples were obtained. We compared two MCMC runs for convergence using random seeds and obtained similar results.

### Electronic supplementary material

Below is the link to the electronic supplementary material.

Supplementary Material 1

Supplementary Material 2

## Data Availability

The 16 newly assembled and annotated CPGs have been submitted to NCBI (https://www. ncbi. nlm. nih. gov), with accession numbers listed in Table S1.Other data generated or analyzed in our study are included in the supplementary files.

## References

[1] Zhang JX, Guan SH, Feng RH, Wang Y, Wu ZY, Zhang YB, Chen XH, Bi KS, Guo DA (2013). Neolignanamides, lignanamides, and other phenolic compounds from the root bark of *Lycium chinense*. J Nat Prod.

[2] Turchetto C, Fagundes NJ, Segatto AL, Kuhlemeier C, Solis Neffa VG, Speranza PR, Bonatto SL, Freitas LB (2014). Diversification in the South American Pampas: the genetic and morphological variation of the widespread *Petunia axillaris* complex (Solanaceae). Mol Ecol.

[3] Zhang ZY, Lu AM, Solanaceae, Wu Z, Raven P (1994). Flora of China.

[4] Chen TY, Jiang XL, Li QS, Zhang ZY, Joongku LE (2012). A new species and a new variety of *Lycium* (Solanaceae) from Ningxia, China. Guihaia.

[5] Liao Q, Wang RJ, Chen TY, Xu L, Jiang (2014). *Lycium Ningxiaense*, a replacement name for Lycium parvifolium. (Solanaceae) Pyhtotaxa.

[6] Li JN, Jiang XL, Li ZG, Chen TY, Zhang ZY (2011). *Lycium qingshuiheense*, a new species of Solanaceae from Ningxia, China. Guihaia.

[7] Xie DM, Zhang XB, Qian D, Zha X, Huang LQ (2016). *Lycium amarum* sp. nov. (Solanaceae) from Xizang, supported from morphological characters and phylogenetic analysis. Nord J Bot.

[8] Potterat O (2010). Goji (*Lycium barbarum* and *L. Chinense*): Phytochemistry, pharmacology and safety in the perspective of traditional uses and recent popularity. Planta Med.

[9] Kim MH, Kim EJ, Choi YY, Hong J, Yang WM (2017). *Lycium chinense* improves post-menopausal obesityvia regulation of PPAR-gamma and estrogen receptor-alpha/beta expressions. Am J Chin Med.

[10] Yao R, Heinrich M, Weckerle CS (2018). The genus *Lycium* as food and medicine: a botanical, ethnobotanical and historical review. J Ethnopharmacology: Interdisciplinary J Devoted Bioscientific Res Indigenous Drugs.

[11] Qin X, Yamauchi R, Aizawa K, Inakuma T, Kato K (2001). Structural features of arabinogalactan-proteins from the fruit of *Lycium chinense*. Carbohydr Res.

[12] Chen X, Zhou J, Cui Y, Wang Y, Duan B, Yao H (2018). Identification of *Ligularia* herbs using the complete chloroplast genome as a super-barcode. Front Pharmacol.

[13] He L, Qian J, Li X, Sun Z, Xu X, Chen S (2017). Complete chloroplast genome of medicinal plant lonicera japonica: genome rearrangement, intron gain and loss, and implications for phylogenetic studies. Molecules.

[14] Zheng GQ, Wang ZZ, Wei JR, Zhao JH, Zhang C, Mi JJ, Zong Y, Liu GH, Wang Y, Xu X, Zeng SH (2024). Fruit development and ripening orchestrating the biosynthesis and regulation of *Lycium barbarum* polysaccharides in goji berry. Int J Biol Macromol.

[15] Bao H, Zheng GQ, Qi GL, Su XL, Wang J (2016). Cellular localization and levels of arabinogalactan proteins in *Lycium barbarum*’s fruit. Pak J Bot.

[16] Zhao JH, Xu YH, Li HX, An W, Yin Y, Wang B, Wang LP, Wang B, Duan LY, Ren YX, Liang XJ, Wang YJ, Wan R, Huang T, Zhang B, Li YL, Luo J, Cao YL. Metabolite-based genome-wide association studies enable the dissection of the genetic bases of flavonoids, betaine and spermidine in wolfberry (*Lycium*). Plant Biotechnol J. 2024, pp:1–18. 10.1111/pbi.14278 PMC11123438 38194521

[17] Zhao JH, Xu YH, Li HX, Zhu XL, Yin Y, Zhang XY, Qin XY, Zhou J, Duan LY, Liang XJ, Huang T, Zhang B, Wan R, Shi ZG, Cao YL, An W (2023). ERF5.1 modulates carotenoid accumulation by interacting with CCD4.1 in *Lycium*. Hortic Res.

[18] Ni LL, Zhao ZL, Lu JN (2016). DNA barcoding construction of medicinal plants in genus *Lycium* L. based on multiple genomic segments. Chin Traditional Herb Drugs.

[19] Hebert PD, Cywinska A, Ball SL, de Waard JR. Biological identifications through DNA barcodes. Proceedings of the Royal Society B: Biological Sciences. 2003;270:313–321. 10.1098/rspb.2002.2218 PMC1691236 12614582

[20] Sanchez-Puerta MV, Abbona CC (2014). The chloroplast genome of *Hyoscyamus niger* and a phylogenetic study of the tribe Hyoscyameae (Solanaceae). PLoS ONE.

[21] Yang Y, Dang Y, Li Q, Lu J, Li X, Wang Y (2014). Complete chloroplast genome sequence of poisonous and medicinal plant Datura stramonium: Organizations and implications for genetic engineering. PLoS ONE.

[22] Shaw J, Lickey EB, Schilling EE, Small RL (2007). Comparison of whole chloroplast genome sequences to choose noncoding regions for phylogenetic studies in angiosperms: the tortoise and the hare III. Am J Bot.

[23] Shaw J, Lickey EB, Beck JT, Farmer SB, Liu W, Miller J, Siripun KC, Winder CT, Schilling EE, Small RL (2005). The tortoise and the hare II: relative utility of 21 noncoding chloroplast DNA sequences for phylogenetic analysis. Am J Bot.

[24] Nielsen AZ, Ziersen B, Jensen K, Lassen LM, Olsen CE, Moller BL, Jensen PE (2013). Redirecting photosynthetic reducing power toward bioactive natural product synthesis. ACS Chem Biol.

[25] Yang ZR, Huang YY, An WL, Zheng XS, Huang S, Liang LL (2019). Sequencing and structural analysis of the complete chloroplast genome of the medicinal plant lyciumchinense mill. Plants.

[26] Guo XY, Liu JQ, Hao GQ, Zhang L, Mao KS, Wang XJ, Zhang D, Ma T, Hu QJ, Al-Shehbaz IA, Koch MA (2017). Plastome phylogeny and early diversification of Brassicaceae. BMC Genomics.

[27] Xie HH, Zhang L, Zhang C, Chang H, Xi ZX, Xu XT (2022). Comparative analysis of the complete chloroplast genomes of six threatened subgenus *Gynopodium* (Magnolia) species. BMC Genomics.

[28] Hu H, Hu QJ, Al-Shehbaz IA, Luo X, Zeng TT, Guo XY, Liu JQ (2016). Species delimitation and interspecific relationships of the genus *Orychophragmus* (Brassicaceae) inferred from whole chloroplast genomes. Front Plant Sci.

[29] Palmer JD (1985). Comparative organization of chloroplast genomes. Annu Rev Genet.

[30] Daniell H, Lin CS, Yu M, Chang WJ (2016). Chloroplast genomes: diversity, evolution, and applications in genetic engineering. Genome Biol.

[31] Wicke S, Schneeweiss GM, dePamphilis CW, Muller KF, Quandt D (2011). The evolution of the plastid chromosome in land plants: gene content, gene order, gene function. Plant Mol Biol.

[32] Kim GB, Lim CE, Kim JS, Kim K, Lee JH, Yu HJ (2020). Comparative chloroplast genome analysis of *Artemisia* (Asteraceae) in East Asia: insights into evolutionary divergence and phylogenomic implications. BMC Genomics.

[33] Xiong Q, Hu YX, Lv WQ, Wang QH, Liu GX, Hu ZY (2021). Chloroplast genomes of five *Oedogonium* species: genome structure, phylogenetic analysis and adaptive evolution. BMC Genomics.

[34] Yang YX, Zhi LQ, Jia Y, Zhong QY, Liu ZL, Yue M (2020). Nucleotide diversity and demographic history of *Pinus bungeana*, an endangered conifer species endemic in China. J Syst Evol.

[35] Zhang FJ, Wang T, Shu XC, Wang N, Zhuang WB, Wang Z (2020). Complete chloroplast genomes and comparative analyses of L. Chinensis, L. Anhuiensis, and L. Aurea (Amaryllidaceae). Int J Mol Sci.

[36] Li HT, Yi TS, Gao LM, Ma PF, Zhang T, Yang JB (2019). Origin of angiosperms and the puzzle of the jurassic gap. Nat Plants.

[37] Cui YX, Zhou JG, Chen XL, Xu ZC, Wang Y, Sun W, Song JY, Yao H (2019). Complete chloroplast genome and comparative analysis of three *Lycium* (Solanaceae) species with medicinal and edible properties. Gene Rep.

[38] Yin XL, Fang KT, Liang YZ, Wong RN, Ha AWY (2005). Assessing phylogenetic relationships of *Lycium* samples using RAPD and entropy theory. Acta Pharmacol Sin.

[39] Xin T, Yao H, Gao H, Zhou X, Ma X, Xu C, Chen J, Han J, Pang X, Xu R (2013). Super food *Lycium barbarum* (solanaceae) traceability via an internal transcribed spacer 2 barcode. Food Res Int.

[40] Ran ZH, Li Z, Xiao X, An MT, Yan C (2024). Complete chloroplast genomes of 13 species of sect. Tuberculata Chang (*Camellia* L.): genomic features, comparative analysis, and phylogenetic relationships. BMC Genomics.

[41] Yang Z, Ma WX, Yang XH, Wang LJ, Zhao TT, Liang LS, Wang GX, Ma QH (2022). Plastome phylogenomics provide new perspective into the phylogeny and evolution of Betulaceae (Fagales). BMC Plant Biol.

[42] Song Y, Wang SJ, Ding YM, Xu J, Li MF, Zhu SF (2017). Chloroplast genomic resource of Paris for species discrimination. Sci Rep.

[43] Cheng H, Li JF, Zhang H, Cai BH, Gao ZH, Qiao YS (2017). The complete chloroplast genome sequence of strawberry (*Fragaria × ananassa* Duch.) And comparison with related species of Rosaceae. PeerJ.

[44] Clegg MT, Gaut BS, Learn GH, Morton BR (1994). Rates and patterns of chloroplast DNA evolution. Proc Natl Acad Sci USA.

[45] Tyagi S, Jung JA, Kim JS, Won SY (2020). Comparative analysis of the complete chloroplast genome of mainland Aster Spathulifolius and other Aster species. Plants.

[46] Wu LL, Wei RX, Yang QW, Zhang ZY (2011). A preliminary study on the hybrid origin of new taxa in *Lycium* (Solanaceae). Guihaia.

[47] Firetti F, Zuntini AR, Gaiarsa JW, Oliveira RS, Lohmann LG, VanSluys MA (2017). Complete chloroplast genome sequences contribute to plant species delimitation: a case study of the Anemopaegma species complex. Am J Bot.

[48] Yu XQ, Drew BT, Yang JB, Gao LM, Li DZ (2017). Comparative chloroplast genomes of eleven *Schima* (Theaceae) species: insights into DNA barcoding and phylogeny. PLoS ONE.

[49] Allen GC, Floresvergara MA, Krasynanski S, Kumar S, Thompson WF (2006). A modified protocol for rapid DNA isolation from plant tissues using cetyltrimethy lammonium bromide. Nat Protoc.

[50] Dierckxsens N, Mardulyn P, Smits G (2017). NOVOPlasty: *de novo* assembly of organelle genomes from whole genome data. Nucleic Acids Res.

[51] Kearse M, Moir R, Wilson A, Stones-Havas S, Cheung M, Sturrock S (2012). Geneious basic: an integrated and extendable desktop software platform for the organization and analysis of sequence data. Bioinformatics.

[52] Huang DI, Cronk QC, Plann (2015). A command-line application for annotating plastome sequences. Appl Plant Sci.

[53] Löytynoja A, Goldman N (2008). Phylogeny-aware gap placement prevents errors in sequence alignment and evolutionary analysis. Science.

[54] Castresana J (2000). Selection of conserved blocks from multiple alignments for their use in phylogenetic analysis. Mol Biol Evol.

[55] Lohse M, Drechsel O, Kahlau S, Bock R (2013). OrganellarGenomeDRAW-a suite of tools for generating physical maps of plastid and mitochondrial genomes and visualizing expression data sets. Nucleic Acids Res.

[56] Sayers EW, Cavanaugh M, Clark K, Ostell J, Pruitt KD, Karsch-Mizrachi I (2020). GenBank Nucleic Acids Res.

[57] Frazer KA, Pachter L, Poliakov A, Rubin EM, Dubchak I (2004). VISTA: computational tools for comparative genomics. Nucleic Acids Res.

[58] Brudno M, Malde S, Poliakov A, Do CB, Couronne O, Dubchak I, Batzoglou S (2003). Glocal alignment: finding rearrangements during alignment. Bioinformatics.

[59] Amiryousefi A, Hyvönen J, Poczai P (2018). IRscope: an online program to visualize the junction sites of chloroplast genomes. Bioinformatics.

[60] Rozas J, Ferrer-Mata A, Sánchez-Delbarrio JC, Guirao-Rico S, Librado P, Ramos-Onsins SE, Sánchez-Gracia A (2017). DnaSP 6: DNA sequence polymorphism analysis of large data sets. Mol Biology Evol.

[61] Stamatakis A (2014). RAxML version 8: a tool for phylogenetic analysis and post-analysis of large phylogenies. Bioinformatics.

[62] Ronquist F, Teslenko M, van der Mark P, Ayres DL, Darling A, Hohna S, Larget Bret, Liu L, Suchard MA, Huelsenbeck JP, Notes A (2012). MrBayes 3.2: efficient bayesian phylogenetic inference and model choice across a large model space. Syst Biol.

[63] Rambaut A. FigTree v1. 4. University of Edinburgh, Edinburgh, UK. 2012. http://tree.bio.ed.ac.uk/software/figtree.

[64] Yang ZH (2007). PAML 4: phylogenetic analysis by maximum likelihood. Mol Biol Evol.

[65] Rannala B, Yang Z (2007). Inferring speciation times under an episodic molecular clock. Syst Biol.

[66] Crepet WL, Nixon KC, Gandolfo MA (2004). Fossil evidenceand phylogeny: the age of major angiosperm clades based on mesofossil and macrofossil evidence from cretaceous deposits. Am J Bot.

[67] Vander BJ (1987). Miocene floras in the Lower Rhenish basin and their ecological interpretation. Rev Palaeobotany Playnology.

[68] Särkinen T, Bohs L, Olmstead RG, Knapp S (2013). A phylogenetic framework for evolutionary study of the nightshades (Solanaceae): a dated 1000-tip tree. BMC Ecol Evol.

