# Supplementary material for: Phylogenetic analysis and divergence time estimation of Lycium species in China based on the chloroplast genomes
Source: BMC Genomics. 2024 Jun 6;25:569. doi: 10.1186/s12864-024-10487-9 (PMC11155141; doi:10.1186/s12864-024-10487-9)

**//Supporting Infomation**

**Figue S1** Phylogenetic tree obtained using the maximum likelihood (ML) methods for the *Lycium* species based on 55 PCGs.

**Figue S2** Phylogenetic tree obtained using the Bayesian inference (BI) methods for the *Lycium* species based on 55 PCGs.

**Figue S3** Phylogenetic tree obtained using the maximum likelihood (ML) methods for the *Lycium* species based on complete CPGs.

**Figue S4** Phylogenetic tree obtained using the Bayesian inference (BI) methods for the *Lycium* species based on complete CPGs.

**Figue S1**


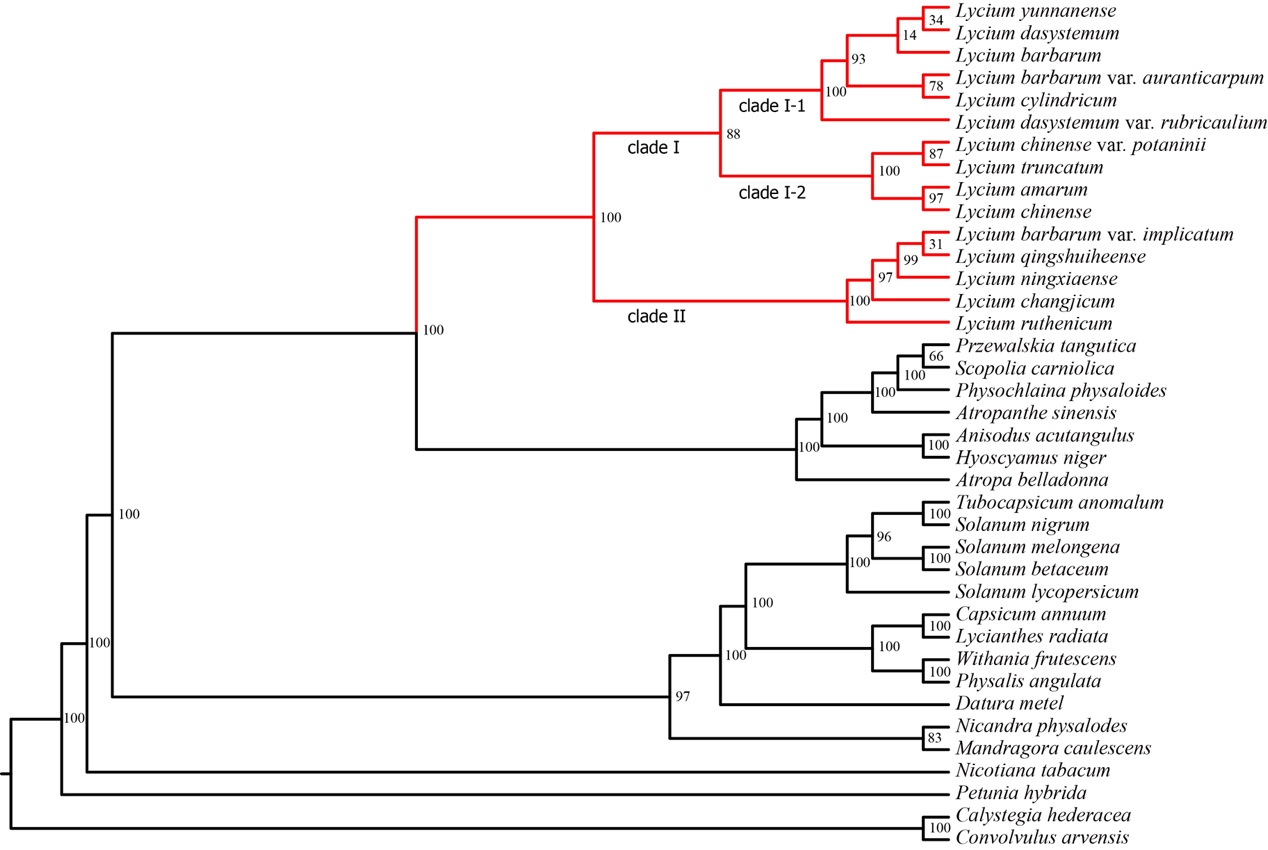


**Figue S2**


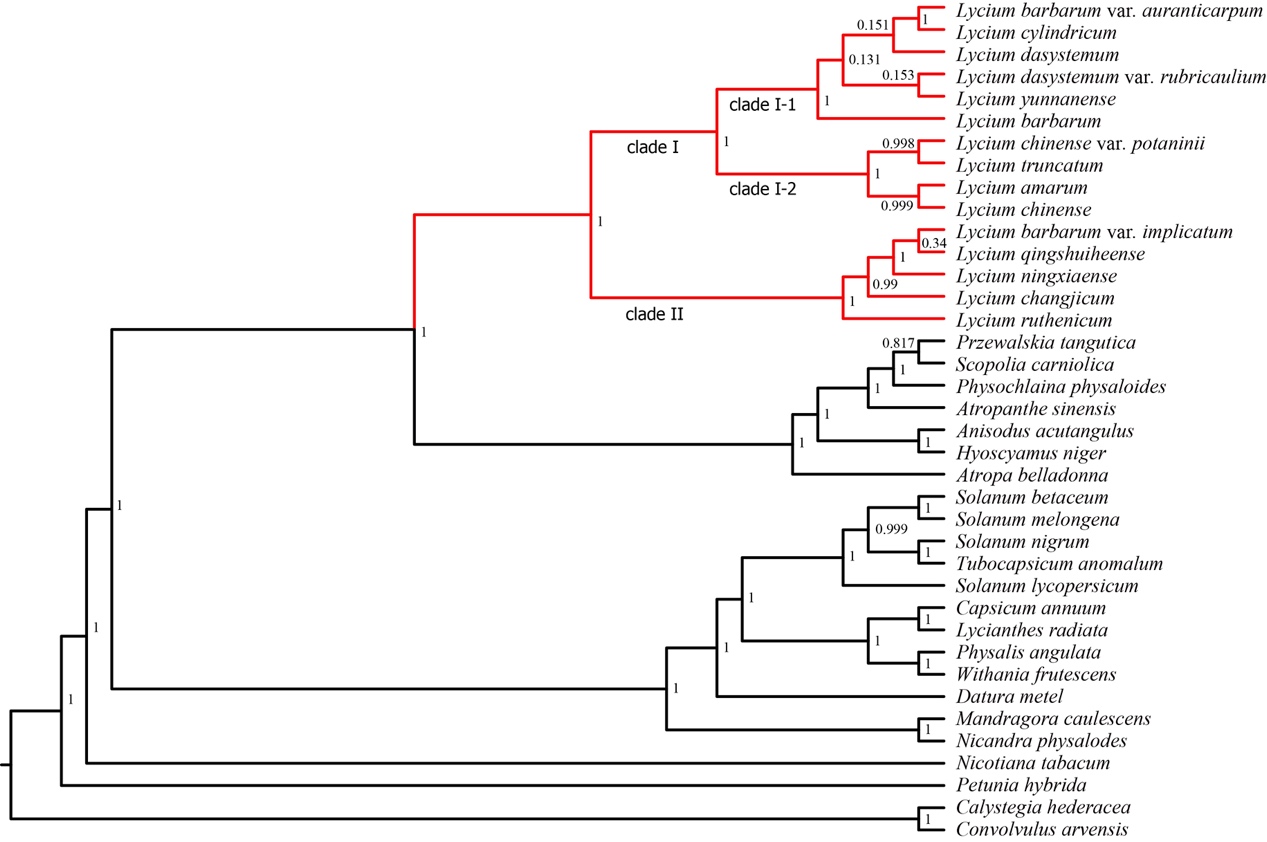


**Figue S3**


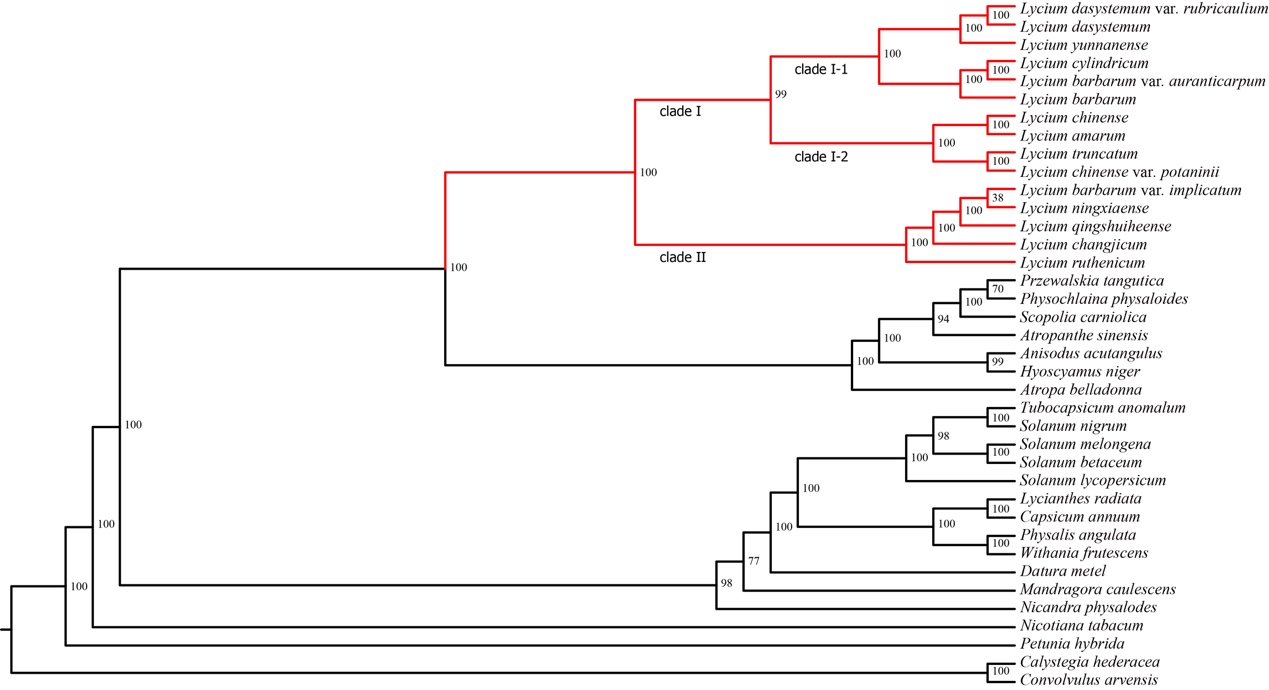


**Figue S4**


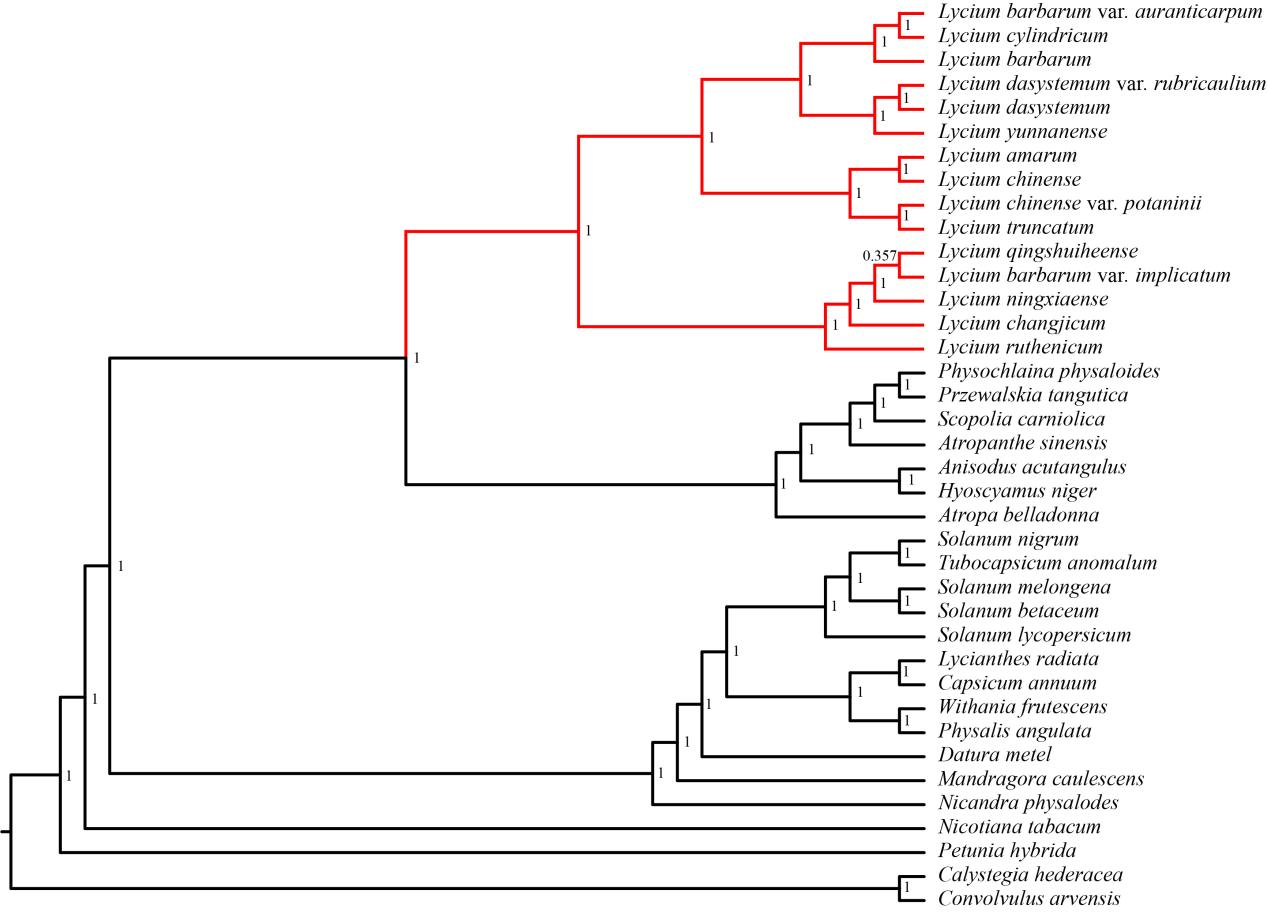

Supplement: Supplementary file 1 — Supplementary Material 1 [file 12864_2024_10487_MOESM1_ESM.docx]
